# Regulation of N6-Methyladenosine after Myocardial Infarction

**DOI:** 10.3390/cells11152271

**Published:** 2022-07-22

**Authors:** Mélanie Vausort, Magdalena Niedolistek, Andrew I. Lumley, Marta Oknińska, Aleksandra Paterek, Michał Mączewski, Xiangyi Dong, Christian Jäger, Carole L. Linster, Przemyslaw Leszek, Yvan Devaux

**Affiliations:** 1 Cardiovascular Research Unit, Luxembourg Institute of Health, 1445 Strassen, Luxembourg; melanie.vausort@lih.lu (M.V.); andrew.lumley@lih.lu (A.I.L.); 2 Department of Medical Biology, Cardinal Wyszynski National Institute of Cardiology, 04-628 Warsaw, Poland; mniedolistek@ikard.pl; 3 Department of Clinical Physiology, Centre of Postgraduate Medical Education, 01-813 Warsaw, Poland; marta.okninska@cmkp.edu.pl (M.O.); aleksandra.paterek@cmkp.edu.pl (A.P.); michal.maczewski@cmkp.edu.pl (M.M.); 4 Luxembourg Centre for Systems Biomedicine, University of Luxembourg, 4367 Belvaux, Luxembourg; xiangyi.dong@uni.lu (X.D.); christian.jaeger@uni.lu (C.J.); carole.linster@uni.lu (C.L.L.); 5 Heart Failure and Transplantology Department, Cardinal Wyszynski National Institute of Cardiology, 04-628 Warsaw, Poland; przemyslaw.leszek@ikard.pl

**Keywords:** m6A, RNA methylation, myocardial infarction, heart failure, biomarker

## Abstract

Development of heart failure (HF) after myocardial infarction (MI) is responsible for premature death. Complex cellular and molecular mechanisms are involved in this process. A number of studies have linked the epitranscriptomic RNA modification N6-methyladenosine (m6A) with HF, but it remains unknown how m6A affects the risk of developing HF after MI. We addressed the regulation of m6A and its demethylase fat mass and obesity-associated (FTO) after MI and their association with HF. Using liquid chromatography coupled to mass spectrometry, we observed an increase of m6A content in the infarcted area of rat hearts subjected to coronary ligation and a decrease in blood. FTO expression measured by quantitative PCR was downregulated in the infarcted hearts. In whole blood samples collected at the time of reperfusion in MI patients, m6A content was lower in patients who developed HF as attested by a 4-month ejection fraction (EF) of ≤40% as compared to patients who did not develop HF (EF > 50%). M6A content was higher in females. These results show that m6A measured in blood is associated with HF development after MI and motivate further investigation of the potential role of m6A as a novel epitranscriptomics biomarker and therapeutic target of HF.

## 1. Introduction

Cardiovascular disease (CVD) is the most common cause of premature death in males below 70 in Europe. Ischemic heart disease represents the most common manifestation of CVD and is responsible for 38% of deaths due to CVD in females and 44% in males in Europe [1]. Myocardial infarction (MI) represents the main cause of ischemic heart disease and is the most common cause of heart failure (HF) development. Despite the improvement of the medical management post-MI, 20–30% of MI patients continue to develop HF at 1 year after hospital discharge [2]. A challenge resides in the current inability to accurately predict whether a patient will develop HF after MI. Being able to identify patients at high risk of HF at an early stage after MI would allow implementing personalized healthcare, thereby reducing disease burden [3]. Routine biomarkers for HF diagnosis, especially natriuretic peptides such as N-terminal pro-brain natriuretic peptide (NT-proBNP), are useful but show some limitations for HF prognostication post-MI due to fluctuations of their circulating levels during the first few days after MI [4]. Therefore, there is a clinical need for new biomarkers to help in risk stratification after MI.

Heart failure often develops as a consequence of left ventricular remodelling after MI, which is a complex process involving both molecular and cellular pathways. A deeper understanding of these pathways could form the basis for the discovery of new biomarkers and therapeutic approaches. Here, we focus our attention on a still poorly characterized molecular epitranscriptomic mechanism named N6-methyladenosine (m6A) RNA methylation.

M6A RNA methylation is the most prevalent, abundant and reversible epitranscriptomic modification in mammalians. It plays important roles in the regulation of many physiological processes [5,6,7,8] and pathologies including cancer [9,10], neurodegenerative [11,12] and cardiovascular diseases [13,14,15]. Identified in the 1970’s in eukaryote messenger RNAs (mRNAs) [16], m6A was recently identified in other types of RNA such as long non-coding RNAs, circular RNAs and small RNAs [17,18]. Two protein families mediate the reversible state of m6A in RNA. On one hand, the m6A writer protein complex includes several proteins such as methyltransferase like 3 (METTL3), methyltransferase like 14 (METTL14), Wilms tumor 1 associating protein (WTAP/KIAA0105) and Vir like m6A methyltransferase associated (VIRMA). Among this m6A writer complex, METTL3 and METTL14 are mainly responsible for the m6A catalytic activity by transferring a methyl group to adenosine on a target RNA molecule [5,9]. On the other hand, the demethylation activity (i.e., the removal of the methyl group from m6A) is mainly controlled by two independent demethylases: fat mass and obesity-associated (FTO) and AlkB homolog 5, RNA demethylase (ALKBH5). M6A is predominantly found in the 3′ untranslated region of mRNAs [19,20]. Functionally, m6A can impact gene expression in different ways, for instance by regulating pre-mRNA processing, mRNA nuclear export, mRNA stability or translation efficiency [5,21]. Similarly, m6A in long non-coding RNAs and microRNAs can modulate their transcript levels, biogenesis and biological functions [17,18].

Several studies have shown that m6A and its regulators are modulated during CVD. For instance, the m6A demethylase FTO modulates cardiac m6A levels in heart disease [22,23,24]. During hypertrophy and HF development after transverse aortic constriction in a Fto-knockout mouse model as well as in human end-stage HF, m6A levels were found to be altered in cardiac tissue and affect structural plasticity and cardiometabolic function [22]. A decrease of FTO expression as well as an increase of the global m6A content was observed in failing hearts from pig and mouse models in addition to human failing hearts [23,24]. FTO overexpression attenuates the ischemia-induced increase of m6A and the cardiac contractile function in failing mouse hearts [24]. M6A regulators are modulated between idiopathic or ischemic MI patients vs. control hearts [25]. In peripheral blood samples of patients with HF with preserved ejection fraction, expression levels of some m6A regulators including FTO were upregulated compared to healthy controls [26]. Together, these studies support a role for m6A and FTO in HF development. However, whether m6A levels in blood cells are associated with the development of HF after MI is still unknown. This could become relevant for the design of novel prognostic biomarkers and therapeutic targets. Here, we analysed m6A and FTO levels in rats and humans after MI and evaluated, for the first time, the potential of m6A measured in blood as a new HF biomarker.

## 2. Materials and Methods

### 2.1. Cell Culture Experiments

The SH-SY5Y neuroblastoma cell line (ATCC, LGC Standards, Molsheim, France) was cultured in Dulbecco’s Modified Eagle’s Medium (Lonza, Verviers, Belgium) with 10% fetal bovine serum (Lonza, Verviers, Belgium) under 95% air/5% CO_2_ atmosphere at 37 °C. For overexpression of FTO, a DNA sequence coding for human FTO (CDS from NM_001082432) was cloned into a pcDNA3.1 vector (ThermoFisher Scientific, Merelbeke, Belgium). Cells were transfected with pcDNA3.1_FTO plasmid or pcDNA3.1 empty vector as mock control using Lipofectamine 2000 (ThermoFisher Scientific, Merelbeke, Belgium). For silencing, RNAimax transfection reagent (ThermoFisher Scientific, Merelbeke, Belgium) was used to transfect SH-SY5Y cells with a commercially available anti-FTO siRNA (10 nM; SI04293625, Hs_FTO_7 FlexiTube siRNA, Qiagen, Venlo, Netherlands) or AllStars siRNA as negative control. After 48 h, cells were collected in 1× cell lysis buffer (Cell Signaling Technology, Bioké, Leiden, The Netherlands) for total protein isolation or in QIAzol lysis reagent (Qiagen, Venlo, The Netherlands) for RNA extraction. All samples were stored at −80 °C until usage.

### 2.2. Rat Experiments

Animal procedures were in conformity with the guidelines from Directive 2010/63/EU of the European Parliament on the protection of animals used for scientific purposes and the RRIVE guidelines. The study was approved by the local ethics committee (Second Warsaw Local Ethics Committee for Animal Experimentation, WAW2/031/2018, 23 February 2018). The animals were housed in an air-conditioned facility with a controlled temperature (21 ± 1 °C) and humidity (55 ± 5%) and maintained on a 12 h artificial dark-light cycle (lights off at 07:00 A.M.) with food (regular rat chow) and water available ad libitum.

Ten Wistar rats (male, 320–350 g) were used in the study; six rats underwent coronary ligation to induce MI, and four rats underwent sham operation. As described [27], rats were anaesthetized with ketamine HCl (100 mg/kg bodyweight, intraperitoneal) and xylazine (5 mg/kg bodyweight, intraperitoneal) and left thoracotomy was performed. The heart was externalized, and a suture (5-0 silk) was placed around the proximal left coronary artery and tightly tied. The heart was internalized, the chest was closed, and the pneumothorax was reduced. Sham-operated rats were subjected to the same protocol, except that the snare was not tied. After 1 h, rats underwent echocardiography and were sacrificed, whole blood samples were collected from the heart in PAXgene™ blood RNA tubes (Becton Dickinson, Warszawa, Poland), and heart tissues were collected. The infarcted area of the left ventricle was separated from the non-infarcted (remote) area, and tissue samples were quickly frozen in liquid nitrogen and stored at −80 °C. PAXgene™; blood RNA tubes were also stored at −80 °C. 

Echocardiography was performed using MyLab25 (Esaote, Genova, Italy) with 13 MHz linear array transducer, as described [28]. Under light isoflurane anesthesia, left ventricular end-diastolic and end-systolic areas (LVEDA and LVESA, respectively) were determined from the long-axis view at the aortic valve level, and ejection fraction was calculated as (LVEDA − LVESA) × 100%/LVEDA. Regional LV wall motion abnormalities were assessed using the wall motion index (WMI). The contractility of 12 wall segments visualized in the midpapillary short-axis view and 11 segments visualized in the long-axis view was graded as 1 (normal) or 0 (abnormal), and the total WMI was calculated. The normal hearts had WMI  =  23. Our previous results [28] revealed that WMI closely correlated with infarct size and that WMI  =  15 corresponded to infarct size ~40%, and thus infarct size was estimated from WMI data. 

### 2.3. Human Samples

Blood samples from twelve patients of the Luxembourg Acute Myocardial Infarction Registry completed at the Institut National de Chirurgie Cardiaque et de Cardiologie Interventionnelle and the Department of Cardiology of the Centre Hospitalier de Luxembourg were used in this study [29]. The protocol has been approved by the ethics committee of Luxembourg (approval CNER 201001/09 from 29 December 2009). All patients signed an informed consent. All patients had an acute MI and were treated with primary percutaneous coronary intervention. Blood samples were withdrawn at the time of reperfusion via an arterial catheter into PAXgene™ blood RNA tubes (Becton Dickinson, Aalst, Belgium) and were stored at −80 °C. Left ventricular ejection fraction (EF) was determined after 4 months using echocardiography. Six patients developed left ventricular dysfunction as attested by a 4-month EF ≤ 40%, and 6 patients had a preserved EF (>50%). Patients in both groups were matched according to age and sex. 

### 2.4. Total RNA Extraction

Total RNA was isolated from cultured cells or rat cardiac tissue samples using the miRNeasy mini kit (Qiagen, Venlo, The Netherlands) according to the manufacturer’s instructions. For rat and human blood samples collected in PAXgene™ RNA tubes, the PAXgene™ Blood RNA Kit (Qiagen, Venlo, The Netherlands) was used to extract total RNA. An on-column RNase-free DNase I (Qiagen, Venlo, The Netherlands) step was applied to eliminate potential genomic DNA contamination. RNA quantity was measured using a NanoDrop 1000 spectrophotometer (Isogen; Utrecht, The Netherlands). 

### 2.5. RNA Preparation for m6A LC-MS Measurement

Five hundred nanograms of total RNA were used per sample to measure m6A by LC-MS. A description of the RNA treatment and the LC-MS set-up is available in the Supplementary Materials. A range from 1 to 10 ng/mL of m6A standards were used to generate a calibration curve (Figure S1). 

### 2.6. m6A RNA Methylation Quantification Assay (Colorimetric ELISA)

An m6A RNA methylation quantification assay (P-9005, EpiGentek, Gentaur, Kampenhout, Belgium) was used to measure m6A in RNA samples from rats. Briefly, 300 ng of total RNA were used per sample. All samples were tested in duplicates. A standard curve with a range from 0.02 ng to 1 ng of m6A per well was used as recommended by the manufacturer and allowed to calculate the amount (ng) of m6A present in samples.

### 2.7. Reverse-Transcription and Quantitative Polymerase Chain Reaction (RT-qPCR)

Reverse transcription was performed using 1µg of total RNA and SuperScript II Reverse Transcriptase (Invitrogen, ThermoFisher Scientific, Merelbeke, Belgium) as described by the manufacturer. Controls without reverse transcriptase were performed to ensure the absence of genomic DNA amplification during PCR reactions. CFX96 Real-Time PCR was performed with IQ SYBR Green supermix (Bio-Rad, Temse, Belgium) and primers designed using the Beacon Designer software (PREMIER Biosoft, Version 8.20, San Francisco, CA, USA; Fto-forward 5′-AGGCAGTTCTGGTTTCAAGGA-3′; Fto-reverse 5′-TTCACGAAGCACGGCATTTG-3′). 18S RNA was used as normalizer and was assessed using the following primers [30]: 18s-forward 5′-CATTCGAACGTCTGCCCTAT-3′; 18s-reverse 5′-GTTTCTCAGGCTCCCTCTCC-3′. PCR conditions were as follows: 3 min at 95 °C, 30 s at 95 °C, and 1 min at 58 °C for FTO or 64 °C for 18 s (40-fold). PCR specificity was confirmed by the presence of a single peak in the melting curve analysis and by Sanger sequencing of the PCR amplicon using the BigDye™ Terminator v1.1 kit (Applied Biosystems, ThermoFisher Scientific, Merelbeke, Belgium). Relative expression levels (2-ΔCq) were calculated using 18S as housekeeping gene and the CFX Maestro 2.2 software (Bio-Rad, Temse, Belgium).

### 2.8. FTO Protein Quantification by Western Blot

Concentration of total proteins in cell samples was determined using a BCA protein assay kit (Pierce, ThermoFisher Scientific, Merelbeke, Belgium). For each sample, 10 µg of proteins were loaded on a 10% polyacrylamide gel. After transfer to an PVDF membrane, No-Stain^TM^ protein labeling reagent (ThermoFisher Scientific, Merelbeke, Belgium) was applied for total protein normalization. The membrane was blocked for 1 h in 1X Tris-buffered saline-0.1% Tween^®^ 20 (VWR; Leuven, Belgium) containing 5% bovine serum albumin (VWR; Leuven, Belgium) and was incubated overnight at 4 °C with an anti-human FTO antibody (clone 5-2H10; 1/1000 dilution, Merck Millipore, Hoeilaart, Belgium). After washing, anti-mouse antibody coupled to HRP (Jackson ImmunoResearch, Ely, UK) and SuperSignal WestDura Extended Duration Substrate (Pierce, ThermoFisher Scientific, Merelbeke, Belgium) were used for revelation. Pictures were captured using IBright^TM^ FL1500 Imaging system (ThermoFisher Scientific, Merelbeke, Belgium) and analyzed using iBright Analysis Software (V4.0.0, ThermoFisher Scientific, Merelbeke, Belgium). Unprocessed original images of blots are shown in the Supplementary Materials (Figure S3).

### 2.9. Statistical Analysis

Comparisons between two groups were achieved using a two-tailed *t*-test or Wilcoxon-Mann-Whitney test for continuous variables and Fisher’s exact test for categorical variables. Correlations were evaluated using the Spearman rank correlation test. *p*-values ≤ 0.05 were considered significant. SigmaPlot software (V14.5, Systat, Palo Alto, CA, USA) was used for statistical analysis.

## 3. Results

### 3.1. Validation of the Measurement of m6A by LC-MS

**Figure 1:**
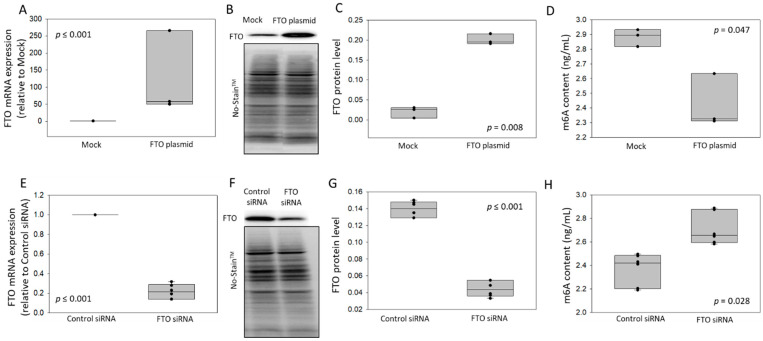
Gain- and loss-of-function experiments to validate m6A detection by LC-MS. SH-SY5Y cells were transfected for 48 h with a FTO over-expressing plasmid (**A**–**D**) or FTO siRNA (**E**–**H**). FTO mRNA levels were measured by RT-qPCR and normalized by 18S. Relative FTO expression was calculated versus mock (**A**, *n* = 3) or control siRNA (**E**, *n* = 6). FTO protein was quantified by Western blot, and No-Stain^TM^ Total Protein Normalization was applied for relative protein level calculation: representative experiments (**B**,**F**; unprocessed original blot images are provided in Supplementary Figure S3) and quantification (**C**, *n* = 3 and **G**, *n* = 6). M6A levels (ng/mL) were assessed by LC-MS (**D**, *n* = 3 and **H**, *n* = 6). *p*-values from 2-group comparisons are shown.

We developed and validated an LC-MS-based method for the quantification of m6A (see Section 2.5 and Supplementary Materials for technical details). First, method linearity was verified by analysis of a calibration curve with an acceptable correlation coefficient of R = 0.986 (Supplementary Figure S1). The calibrated range of the method was from 1 to 10 ng/mL. Accuracy (between 90.6 and 98.3%) and precision (between 9.7 and 22.9%) for the entire procedure were assessed at three different concentrations (QC samples: 3, 5 and 9 ng/mL). To evaluate the ability of the method to detect m6A changes, we conducted gain- and loss-of function experiments in cultured SH-SY5Y cells. We overexpressed FTO using a plasmid and silenced it using siRNA. After a total of 48 h post-transfection by the FTO plasmid, we observed an up-regulation of FTO mRNA (Figure 1A) and protein (Figure 1B,C) levels compared to the empty plasmid control. On the other hand, 48 h after transfection with FTO siRNA, we observed a decrease of FTO mRNA (Figure 1E) and protein (Figure 1F,G) compared to control siRNA. These results attested the desired modulation of m6A demethylase FTO. M6A levels were then measured by LC-MS in total RNA samples extracted from these cells (500 ng RNA aliquots were used for each m6A measurement). We observed that m6A levels were decreased upon FTO overexpression (Figure 1D) and increased upon FTO silencing (Figure 1H). These results confirmed first that modulation of FTO induces changes in m6A levels and second that the implemented methodology is able to detect variations in m6A levels within the measuring interval.

### 3.2. Regulation of m6A Levels and FTO Expression after Coronary Ligation in Rats

**Figure 2:**
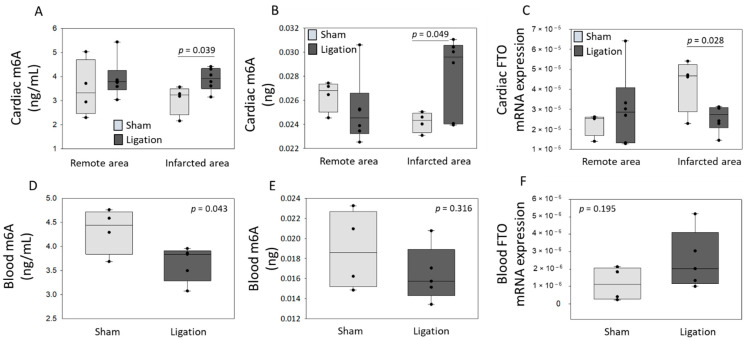
M6A levels and FTO mRNA expression in a rat model of myocardial infarction. Rats were subjected to coronary ligation or sham-operation. After 1 h, rats were sacrificed, cardiac tissue (*n* = 6 after ligation and *n* = 4 sham-operated) and blood samples (*n* = 5 after ligation and *n* = 4 sham-operated) were harvested, and remote and infarcted areas of the heart were dissected before storage. Total RNA was extracted from tissue (**A**–**C**) and blood (**D**–**F**) samples. M6A levels were assessed by LC-MS (**A**,**D**; ng/mL) and by a colorimetric ELISA (**B**,**E**; ng of m6A contained in 300 ng total RNA). FTO mRNA levels were measured by RT-qPCR and normalized by 18S (**C**,**F**). *p*-values for two-group comparisons are indicated.

To investigate the regulation of m6A levels and FTO expression after MI, we subjected rats to coronary ligation or sham-operation. Representative echocardiographic images and videos performed 1 h after surgery are shown (Figure S2A and Supplementary Videos: Movie 1-sham and Movie 2-MI). Left ventricular end-diastolic (Figure S2B) and end-systolic (Figure S2C) areas measured by echocardiography were both increased after coronary ligation. Calculated ejection fraction (Figure S2D) was reduced 1 h after coronary ligation. These data support our model of MI in rat. Left ventricular tissue and whole blood samples were collected 1 h after surgery, and the infarcted and remote areas of the heart were dissected and separately stored for further analysis. Total RNA was extracted from all tissue and blood samples; m6A levels were quantified by LC-MS and by a colorimetric ELISA. FTO expression was assessed by quantitative PCR. M6A levels were increased in the infarcted area after coronary ligation as compared to sham-operated rats and remained unchanged in the remote zone. These results are consistent between LC-MS and ELISA measurements (Figure 2A,B). In blood samples, m6A content was decreased in rats subjected to coronary ligation as compared to sham-operated rats using LC-MS (Figure 2D). Using ELISA, m6A content showed a similar pattern, although not reaching statistical significance (Figure 2E). Conversely, FTO expression was decreased in the infarct zone and showed a trend to increase in blood samples after coronary ligation (Figure 2C,F). Therefore, we observed an inverse relationship between m6A and FTO regulation at an early stage after MI, both in the infarcted area of the heart and in blood samples.

### 3.3. Association between m6A Levels in Blood of Patients after MI and Cardiac Dysfunction

To evaluate a potential association between m6A and the development of HF after MI in humans, we conducted a pilot study using whole blood samples collected at the time of reperfusion in 12 acute MI patients. Six patients developed HF, as attested by a 4-month ejection fraction (EF) ≤ 40%, and six patients did not develop HF (4-month EF > 50%). These two groups were sex- and age-matched and had similar demographic and clinical data, except for peak levels of creatine phosphokinase (CPK) and cardiac troponin T (cTnT) which, as expected, were higher in patients developing HF (Table 1).

**Figure 3:**
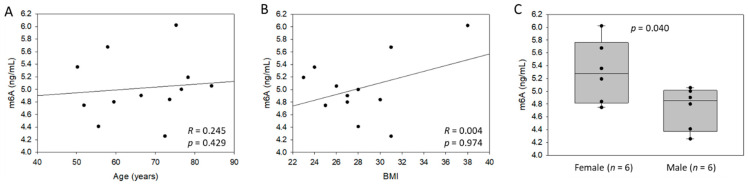
M6A levels in the blood of 12 patients after myocardial infarction (MI): association with age (**A**), body mass index (BMI) (**B**) and gender (**C**). M6A content was assessed by LC-MS using total RNA extracted from whole blood samples. Spearman correlation coefficients and *p*-value are indicated.

Total RNA isolated from whole blood samples was used for M6A determination by LC-MS. As shown in Figure 3A,B, the m6A content was not associated with age and body mass index in these patients. Interestingly, female patients had a higher m6A content in the blood after MI compared to males (Figure 3C).

**Figure 4:**
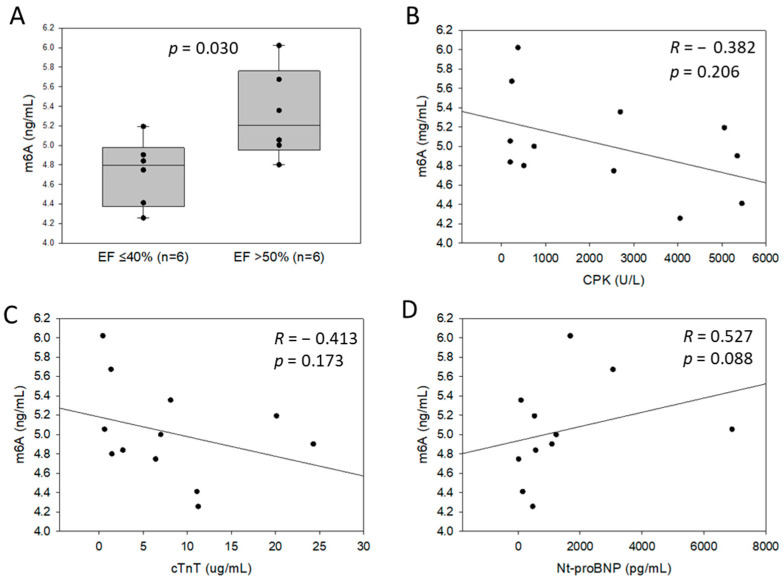
M6A levels in the blood of 12 patients after myocardial infarction (MI): association with ejection fraction (EF) (**A**), peak levels of cardiac markers CPK (**B**), cTnT (**C**), and admission levels of Nt-proBNP (**D**). Six patients developing heart failure 4 months after MI (EF ≤ 40%), and 6 patients with preserved EF (>50%) were included. M6A levels in total RNA extracted from whole blood samples collected at the time of reperfusion were quantified by LC-MS. Spearman correlation coefficients and *p*-value are indicated.

Patients developing HF 4-month after MI (EF ≤ 40%) had lower m6A levels in the blood as compared to patients with a preserved EF (>50%) (Figure 4A). M6A levels were not correlated with the peak levels of cardiac injury markers CPK and cTnT (Figure 4B,C) but showed a trend towards a positive correlation with NT-proBNP levels measured at admission (Figure 4D). Comorbidities such as diabetes mellitus, hypertension, hypercholesterolemia, tobacco consumption and previous MI history were not associated with blood m6A levels in this group of patients (data not shown).

## 4. Discussion

We report the implementation of an LC-MS-based method which was used to quantify m6A levels in cardiac tissue and blood samples. We observed increased m6A levels in the infarcted area of rat hearts after MI induction and, conversely, a decrease of m6A levels in blood samples. This was paralleled by opposite regulations of the m6A demethylase FTO. In whole blood samples from MI patients, m6A levels were reduced in patients developing HF with a reduced EF after 4 months. Hence, we report a regulation of m6A levels associated with cardiac dysfunction after MI.

M6A RNA methylation is an epitranscriptomics modification known to be implicated in heart development, regeneration and disease [31]. FTO, an m6A demethylase, was identified as the key m6A regulator in cardiovascular disease, notably in cardiac remodelling post MI [22,24,32,33,34]. Several methodologies have been implemented to quantify m6A levels in RNA samples [17]. Some of these methods help to identify and localize specific m6A sites in RNA such as Me-RIP sequencing or m6A-CLIP. Others are used to quantify the global m6A abundance in RNA samples such as dot blot or enzyme-linked immunosorbent assay. Yet, some methods used to quantify the global m6A content lack of reproducibility and accuracy. According to [5,35], LC-MS is a powerful method to specifically quantify m6A with high sensitivity and monitor m6A dynamics, and it has been validated for m6A measurement in comparison to m6A dot blot or 2D-TLC [36,37]. Therefore, an LC-MS method has been implemented for m6A measurements in this study. As described, m6A measurements showed satisfactory reproducibility. Interestingly, we observed an inverse relationship between FTO and m6A levels in experiments using cultured cells, which was consistent with the m6A demethylase function of FTO. Additionally, similar m6A patterns were observed by LC-MS and a commercial colorimetric ELISA. Compared to LC-MS, very low m6A contents were obtained from blood samples of rats using ELISA, and they were sometimes below the first point of the standard curve. Contrarily to LC-MS, the ELISA detects m6A in RNA molecules longer than 70 nucleotides; hence, it could underestimate the global m6A content by exclusion of m6A from small RNAs such as microRNAs. As described [38,39], m6A methylation of microRNAs is present and important for microRNA biogenesis and function. These results validate and motivate the use of LC-MS for its ability to detect changes in m6A levels in total RNA derived from complex biological samples.

Recently, blood levels of m6A have been described as a novel prognostic biomarker in cancer [40,41]. Whether this holds true for cardiovascular disease still needs to be thoroughly tested. In this study, m6A levels and FTO expression were measured in heart and blood samples from a rat MI model. The observed inverse regulation of FTO and m6A in the cardiac tissue and blood suggests a role of the FTO-m6A pathway after MI, as reported previously [24]. Interestingly, the regulation of FTO and m6A were limited to the infarcted area of the hearts and remained unchanged in the remote zone. This is consistent with a specific regulation induced by the stress provoked by coronary ligation and is in line with previous studies in mice and pigs [24].

To investigate the potential of m6A to be used as a prognostic biomarker after MI, we conducted a pilot study using peripheral blood samples of a small group of 12 MI patients. Interestingly, lower m6A levels were observed in the blood of MI patients who developed HF with a reduced 4-month EF as compared to patients who had a preserved EF. These encouraging results highlight the potential of blood m6A levels to be used as an early predictive biomarker of HF development after MI. This is of clinical relevance, since biomarkers able to accurately predict left ventricular remodelling leading to HF after MI are still lacking. The functional association between the regulation of m6A levels in blood and cardiac dysfunction remains to be established, yet our data showing a positive correlation with cardiac stress and HF biomarker NT-proBNP support a link between left ventricular remodelling and RNA methylation. The absence of correlation with the peak levels of CPK and cTnT suggests that m6A regulation is not merely associated with the extent of cardiac damage.

In our group of MI patients, females had higher m6A levels in blood as compared to males. This suggests that there might be different m6A patterns between males and females, which is supported by the implication of m6A in sex determination, as previously reported [42,43,44]. The influence of sex on blood m6A levels and their biomarker potential should be further investigated.

M6A is not the only RNA modification characterized so far, and it will be informative to further characterize the regulation of the different RNA modifications and epitranscriptomics mechanisms associated or playing a role in HF development. This may lead to the discovery of novel biomarkers and therapeutic targets.

Our study is limited by low sample size. Further studies, including a larger number of rats, are needed to elucidate the implication of m6A in the pathophysiology of MI and the development of heart failure. Larger cohorts of patients suffering of MI could be used for additional investigations to strengthen the results highlighted in this pilot study. Furthermore, the identification of the exact genes that are specifically m6A modulated could lead to a potential m6A biomarker signature after MI and help elucidate the mechanisms leading to heart failure development. This could further aid in uncovering the role of m6A in the pathophysiology of heart failure and provide novel avenues for biomarker research in order to improve heart failure prognosis, find new therapeutic targets and drive personalized healthcare.

In conclusion, we report that m6A RNA modification levels are modulated in peripheral blood after MI and may hold some potential as a novel biomarker of HF development. Our data need to be confirmed in larger populations. Whether m6A regulation plays a functional role in left ventricular remodelling remains an open question.

## Figures and Tables

**Table 1:** Demographic and clinical characteristics of two groups of MI patients used for blood M6A quantification. *p*-values for two-group comparisons are indicated.

	Heart Failure EF ≤ 40% *n* = 6		Preserved EF > 50% *n* = 6		*p*-Value
Age, median (range), y	69	(56–78)	67	(50–84)	0.896
Body mass index, median (range)	28	(22–31)	28	(24–38)	0.499
Gender, male, *n* (%)	3	(50)	3	(50)	1.000
Blood cell counts at admission, median (range)					
White blood cells, ×10^9^/L	10.18	(3.66–19.91)	9.22	(3.10–12.98)	0.423
Neutrophiles, ×10^9^/L	7.99	(1.86–16.66)	6.77	(2.43–8.63)	0.818
Lymphocytes, ×10^9^/L	1.42	(0.71–2.65)	1.78	(0.44–4.36)	0.727
Monocytes, ×10^9^/L	0.44	(0.33–1.29)	0.73	(0.16–1.00)	0.855
Platelets, ×10^9^/L	297	(161–474)	250	(169–304)	0.429
Biomarkers at admission, median (range)					
MMP9, ng/mL	472	(209–1237)	298	(149–547)	0.240
TIMP1, ng/mL	174	(116–363)	102	(79–147)	0.065
NT–proBNP, pg/mL	502	(17–1092)	1683	(94–6906)	0.082
Biomarkers, peak values, median (range)					
CPK, median, range, U/L	4555	(201–5456)	441.5	(201–2697)	0.009
cTnT, median, range, µg/L	11.17	(2.7–24.3)	1 405	(0.43–8.11)	0.026
hsCRP, mg/L	14.7	(2.3–71)	6.9	(1.2–199)	0.421
Medical history, *n* (%)					
Prior MI	1	(17)	1	(17)	1.000
Diabetes	2	(33)	2	(33)	1.000
Hypertension	5	(83)	4	(67)	1.000
Hypercholesterolemia	3	(50)	3	(50)	1.000
Tobacco	3	(50)	2	(33)	1.000
Follow-up EF, median (range), %	36	(25–40)	66	(55–75)	<0.001

## Data Availability

Not applicable.

## Supplementary Materials

The following supporting information can be downloaded at: https://www.mdpi.com/article/10.3390/cells11152271/s1; Total RNA sample treatment and LC-MS protocol for m6A determination; Table S1: specific source settings for m6A measurement using LC-MS; Figure S1: calibration curve using m6A standards by LC-MS; Figure S2: Echocardiographic images, videos and parameters in rats 1 h after surgery; Figure S3: FTO protein quantification by Western Blot; Video S1: Movie 1-sham; Video S2: Movie 2-MI.
