# Supplementary material for: Regulation of N6-Methyladenosine after Myocardial Infarction
Source: Cells. 2022 Jul 22;11(15):2271. doi: 10.3390/cells11152271 (PMC9329994; doi:10.3390/cells11152271)
Supplement: Supplementary file 1 [file cells-11-02271-s001.zip › Supplementary Materials.pdf]

Total RNA sample treatment and LC-MS protocol for m6A determination

Total RNA samples were prepared for m6A measurement by LC-MS. Briefly, 500ng of total RNA were first digested using Nuclease P1 to cut the RNA chains into nucleosides and second, dephosphorylated using Calf Intestinal Alkaline Phosphatase (CIP) as described in [37]. Afterwards, the sample was added to 4 volumes of methanol, containing the internal standard (N6-Methyladenosine-d3, ATCC/LGC Standards; 200 ng/mL). To ensure complete protein precipitation, the sample was thoroughly vortexed and incubated for 20 min at 4 °C under shaking followed by centrifugation at 21 000 g for 10 min. 80 µL of the supernatant were collected and dried in a rotary vacuum concentrator overnight. Before measurement, the dried extract was resuspended in 40 µL of 20 mM ammonium formate in water, pH 3.2 (mobile phase A).

LC-MS analysis was performed using a Shimadzu Nexera XR liquid chromatography system coupled to a Sciex QTrap 4000 mass spectrometer equipped with a Turbo V electrospray ionization source. Chromatography was accomplished using a gradient elution in a Phenomenex Kinetex 2.6 µm EVO C18 column (150 x 2.1 mm) at a flow rate of 0.3 mL/min. The column temperature was maintained at 40 °C.

Mobile phase A consisted of 20 mM ammonium formate in water (pH was adjusted to 3.2), mobile phase B was 100% methanol. 10 µL of the samples were injected. Gradient elution started at 2% B for 6 min, followed by a linear increase to 98% B in 3 min and an isocratic delivery at 98% B for 3.5 min. Then, the solvent composition was changed to starting conditions within 0.1 min and the column was equilibrated for 8.4 min. Total runtime was 20 min for a single sample. Target compounds were measured in positive ion mode.

Mass spectrometric data were acquired with the AB SCIEX Analyst software (Version 1.7.1) and analyzed with MultiQuant (Version 3.0.3). Specific source settings for the measurement of all transitions in multiple reaction monitoring mode are given in Table S1. Target compounds were identified by retention times and ion ratios of two transitions per sample. The data set was normalized by using the response ratio of the integrated peak area of m6A and the integrated peak area of the corresponding internal standard (IS: d3-m6A). Absolute concentrations were determined using calibration curves made of authentic standards from 1 to 10ng/mL.

Table S1: **Specific source settings for m6A measurement using LC-MS.**

Q1 Mass (Da)	Q3 Mass (Da)	Time (msec)	ID	DP (Volts)	EP (Volts)	CE (Volts)	CXP (Volts)
282.046	150.100	100	**m6A**	51.000	10.000	23.000	26.000
285.100	153.400	100	**IS: d3-m6A**	62.000	10.000	27.000	26.000

Figure S1: **M6A calibration curve.**

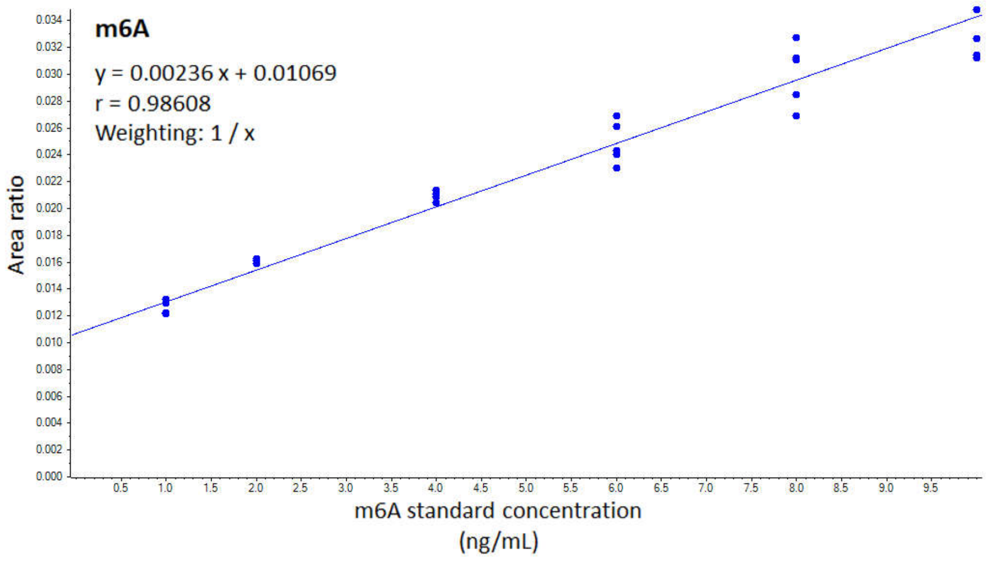

Figure S2: **Echocardiographic images, videos and parameters in rats 1 hour after surgery.** Echocardiography was performed under light isoflurane anaesthesia. Representative echocardiographic left ventricular images and videos from a sham operated rat and rat with myocardial infarction induced by coronary ligation (MI) 1 hour after the procedure (A). Columns I and II show B-mode long axis diastolic and systolic images, respectively. Blue and green lines represent the endocardial surface. Column III includes systolic and diastolic tracings of endocardial surface from columns I and II, respectively. Column IV shows B-mode short axis images, while column V shows corresponding M-mode short axis images. While in the sham operated rat the wall contraction and systolic thickening are fairly uniform, in the MI rat the anterior and lateral wall (upper part of the long axis image, upper and left part of the short axis image and upper part of the M-mode image) is largely non-contractile, corresponding to the ischemic/infarcted region, which results in reduced ejection fraction and increased left ventricular end-systolic area.

Representative videos are available for sham and MI rats (see Supplementary Videos). Video S1 (Movie 1 – sham): Online-only supplementary movie demonstrating left ventricle of sham operated hearts with normal wall contractility in the short axis view. Video S2 (Movie 2 – MI): Online-only supplementary movie demonstrating left ventricle of a rat with a large myocardial infarction; the interventricular septum (on the left) exhibits hypercontractility, while the whole free left ventricular wall (on the right) is akinetic, indicating ischemia/infarction.

Left ventricular end-diastolic area (LVEDA; B), left ventricular end-systolic area (LVESA; C) and, ejection fraction (D) were evaluated for 6 rats subjected to coronary ligation and 4 sham-operated rats. Ejection fraction was calculated as (LVEDA-LVESA)*100%/LVEDA. *p*-values from 2-group comparisons are shown.

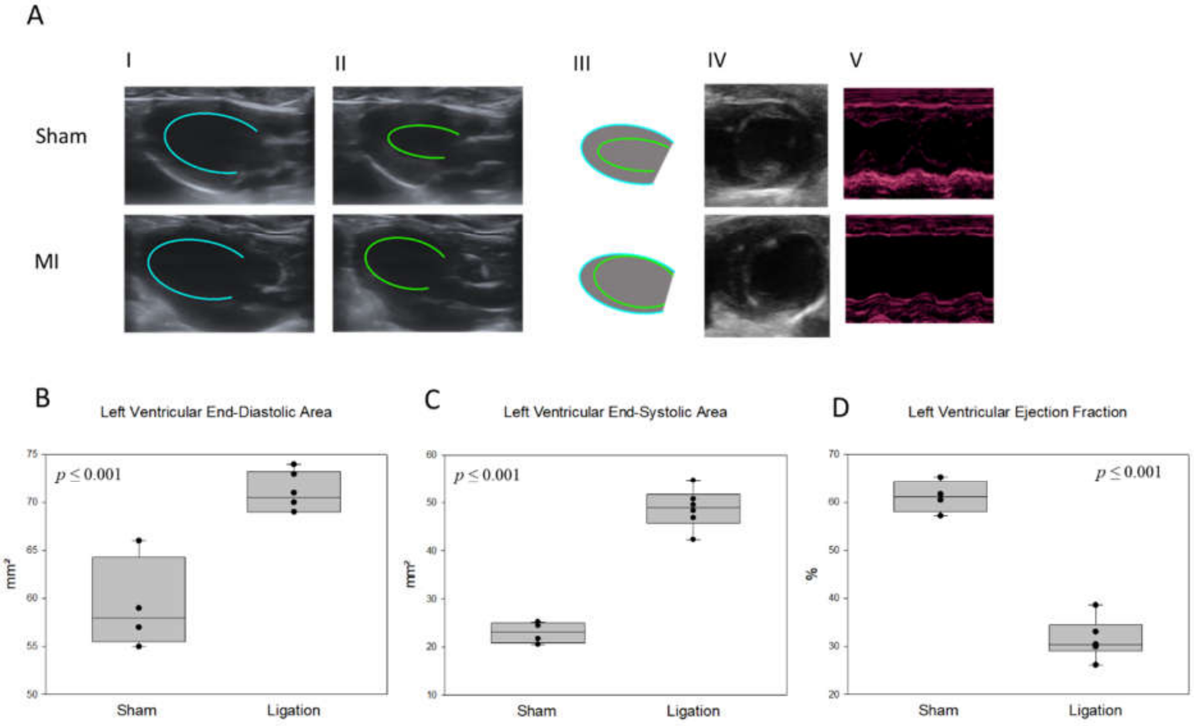

Figure S3: **FTO protein quantification by Western Blot.**

Total proteins from cultured SH-SY5Y cells were harvested 48h after transfection with 100ng of pcDNA3.1_FTO, pcDNA3.1_GFP or mock plasmid (A) or 10nM of FTO or All* negative control siRNA (B). Ten micrograms of proteins were loaded per sample. No-Stain^{TM} protein labeling was applied for total protein normalization. pcDNA 3.1_GFP was used as control for transfection efficiency. Three experiments overexpressing FTO and six experiments silencing FTO were performed. One representative experiment is shown in A and B, respectively.

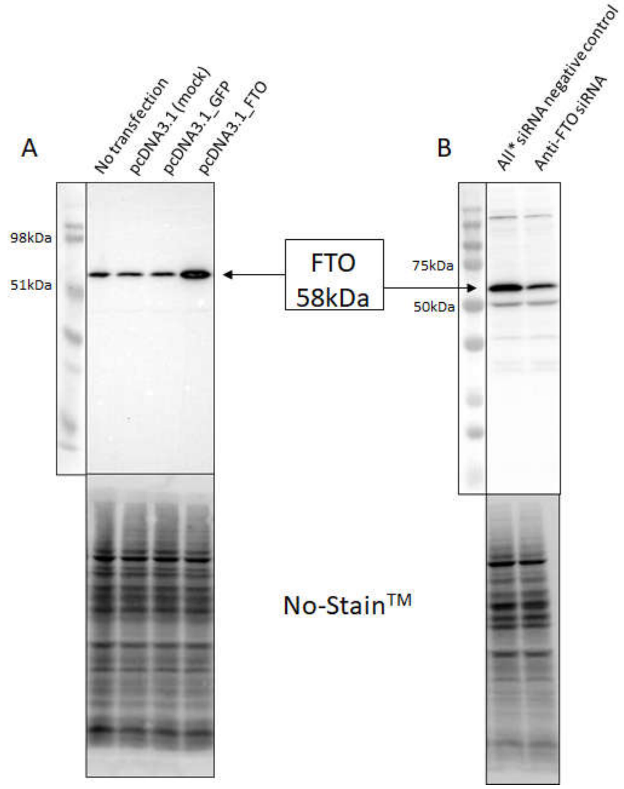

37. Wang, H.; Hu, X.; Huang, M.; Liu, J.; Gu, Y.; Ma, L.; Zhou, Q.; Cao, X. Mettl3-mediated mRNA m(6)A methylation promotes dendritic cell activation. Nat. Commun. 2019, 10, 1898. https://doi.org/10.1038/s41467-019-09903-6.
